# An interpretative review of the wastewater-based surveillance of the SARS-CoV-2: where do we stand on its presence and concern?

**DOI:** 10.3389/fmicb.2024.1338100

**Published:** 2024-01-22

**Authors:** Gayatri Gogoi, Sarangthem Dinamani Singh, Emon Kalyan, Devpratim Koch, Pronami Gogoi, Suman Kshattry, Hridoy Jyoti Mahanta, Md Imran, Rajesh Pandey, Pankaj Bharali

**Affiliations:** ^1^Center for Infectious Diseases, Biological Science and Technology Division, CSIR-North East Institute of Science and Technology (CSIR-NEIST), Jorhat, Assam, India; ^2^Academy of Scientific and Innovative Research (AcSIR), Ghaziabad, India; ^3^Advanced Computation and Data Sciences Division, CSIR-North East Institute of Science and Technology, Jorhat, Assam, India; ^4^Division of Immunology and Infectious Disease Biology, INtegrative GENomics of HOst-PathogEn (INGEN-HOPE) Laboratory, CSIR-Institute of Genomics and Integrative Biology (CSIR-IGIB), New Delhi, India

**Keywords:** SARS-CoV-2, wastewater-based epidemiology, surveillance, next-generation sequencing, complex system

## Abstract

Wastewater-based epidemiology (WBE) has been used for monitoring infectious diseases like polio, hepatitis, etc. since the 1940s. It is also being used for tracking the SARS-CoV-2 at the population level. This article aims to compile and assess the information for the qualitative and quantitative detection of the SARS-CoV-2 in wastewater. Based on the globally published studies, we highlight the importance of monitoring SARS-CoV-2 presence/detection in the wastewater and concurrently emphasize the development of early surveillance techniques. SARS-CoV-2 RNA sheds in the human feces, saliva, sputum and mucus that ultimately reaches to the wastewater and brings viral RNA into it. For the detection of the virus in the wastewater, different detection techniques have been optimized and are in use. These are based on serological, biosensor, targeted PCR, and next generation sequencing for whole genome sequencing or targeted amplicon sequencing. The presence of the SARS-CoV-2 RNA in wastewater could be used as a potential tool for early detection and devising the strategies for eradication of the virus before it is spread in the community. Additionally, with the right and timely understanding of viral behavior in the environment, an accurate and instructive model that leverages WBE-derived data may be created. This might help with the creation of technological tools and doable plans of action to lessen the negative effects of current viral epidemics or future potential outbreaks on public health and the economy. Further work toward whether presence of viral load correlates with its ability to induce infection, still needs evidence. The current increasing incidences of JN.1 variant is a case in point for continued early detection and surveillance, including wastewater.

## Graphical abstract

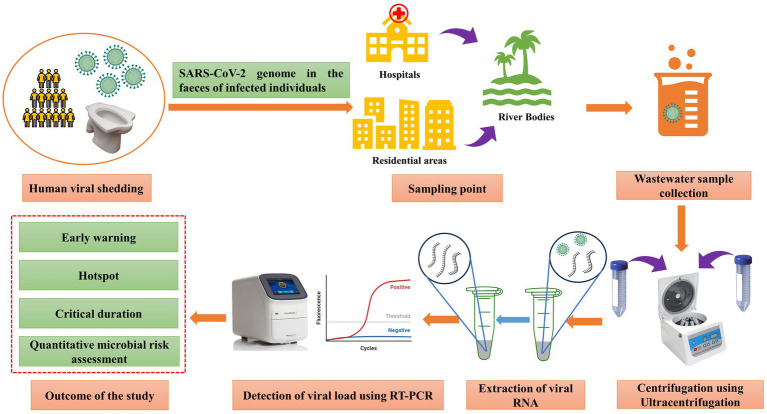

## Introduction

In December 2019, China reported to the World Health Organization (WHO) an epidemic of unknown cause pneumonia in Wuhan, Hubei Province, Central China.^1^ Sequencing of the broncho-alveolar lavage samples by shotgun metagenomics revealed that the pandemic is caused by a novel coronavirus (nCoV) (Chen et al., 2021). Further it was established that the nCoV had a nucleotide resemblance of 75–80% to the severe acute respiratory syndrome coronavirus and thus it was formally categorized as SARS-CoV-2 virus (Ahmed et al., 2020; Zhu et al., 2020). Due to the SARS-CoV-2 mediated COVID-19 pandemic, there has been a paradigm shift in society worldwide which ultimately pose a threat to socioeconomic growth and development (Bisseux et al., 2018; Bhattacharya et al., 2021). SARS-CoV-2 has caused a catastrophic effect on public health, economy, medical infrastructure, supply chain, mental health as well as quality of life in a society globally. This is unparalleled devastation with high number of SARS-CoV-2 infections around the globe (Kumblathan et al., 2022). SARS-CoV-2 levels in the water, wastewater, sludge, and air, as well as on surfaces, are being monitored to estimate the risk of virus infection from the contaminated surroundings. The transmission of SARS-CoV-2 in aerosols has garnered lot of attention, but the assessment of the risk of SARS-CoV-2 spread in wastewater was underestimated and minimum attention has been paid to the danger and necessity of waste disposal as an ecological response to problems related to COVID-19 in water and wastewater environments (Tetteh et al., 2020; Meng et al., 2021). Netherlands, Australia, Japan, India, Italy, Spain, Sweden, Bangladesh, and the United States were among the first nations to report verified occurrences of SARS-CoV-2 RNA in the municipal wastewater. Various discoveries coincided with the first clinically verified cases in these nations. These findings might provide proof of concept for possibly concealed information about the frequency of SARS-CoV-2 infections, as well as a cost-effective means of randomly screening many patients (Kumar, 2021). Clinical samples from a fraction of the symptomatic/hospitalized population throughout the world are being utilized to sequence the SARS-CoV-2 genome using high-throughput sequencing (HTS) methods. This has resulted in the publication of a huge number of genomes (Elbe and Buckland-Merrett, 2017; Shu and McCauley, 2017), and computational tools in genomic epidemiology have offered insight into the origin, dissemination, and variety of these genomes.

**Figure 1:**
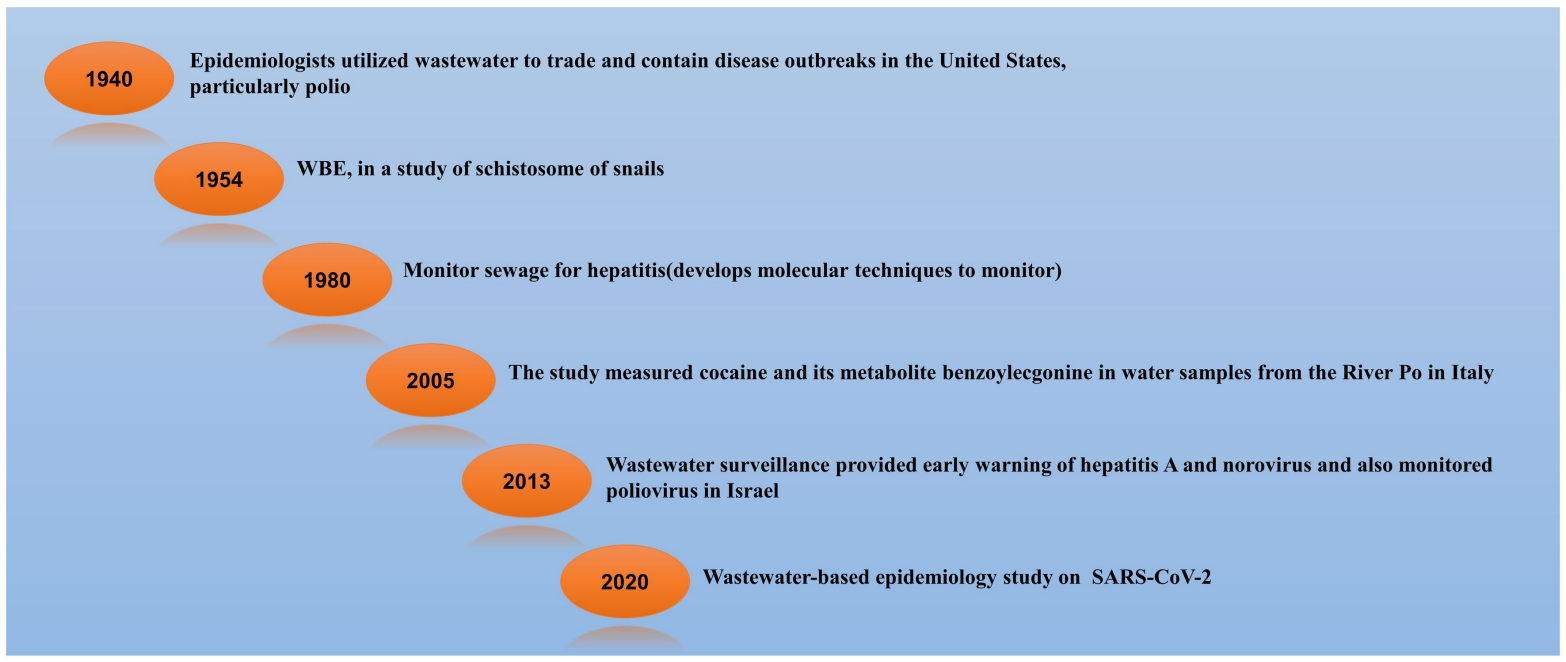
Timeline of Wastewater-Based Epidemiology (WBE) Surveillance. Tracing Pathogens and Monitoring Public Health through Sewage Analysis (Barbosa and Olivier, 1958; Metcalf et al., 1995; Zuccato et al., 2005; Brouwer et al., 2018; Polo et al., 2020; Meng et al., 2021).

The history of wastewater/sewage/WBE (Wastewater Based Epidemiology) started long back. In the 1940s, epidemiologists from the United States utilized wastewater to trade and search for disease outbreaks, particularly polio (Metcalf et al., 1995). WBE in a study of the schistosome of snails started in 1954 in Brazil (Barbosa and Olivier, 1958). After that in 1980, scientists monitored sewage for hepatitis and parallelly developed molecular techniques to monitor the sewage (Meng et al., 2021). From the River Po in Italy, scientists studied cocaine and its metabolite benzoylecgonine in a water sample in 2005 (Zuccato et al., 2005). In 2013, studies were done in Israel and they found norovirus, hepatitis A virus, and poliovirus in the wastewater as early warning surveillance (Brouwer et al., 2018). When the pandemic came in 2020, SARS-CoV-2 wastewater-based surveillance study was started (Polo et al., 2020). The timeline for wastewater/sewage/WBE surveillance is shown in Figure 1.

Wastewater monitoring is relatively economical and effective method of tracking pathogens and estimating the scale and speed of infection in a community (Hart and Halden, 2020). In addition to the respiratory secretions, SARS-CoV-2 sheds into the faces (Kumblathan et al., 2022). Since the domestic wastewater contains bath, shower, and laundry wastewater, it may have the presence of SARS-CoV-2 from the respiratory secretions as well. Detection of SARS-CoV-2 genetic signal in the wastewater through WBE would facilitate the identification of infection existing in a community. In a building, detecting the presence or absence of an infection is helpful to guide mitigation actions, however quantifying the extent (i.e., having a valid method to estimate the number of cases) might allow local governments to better adjust/optimize policy based on the severity of the epidemic (McMahan et al., 2021). Certain viral strains detected in the wastewater have been linked to those discovered in different clinical samples using WBE (Carducci et al., 2006; Weil et al., 2017; Bisseux et al., 2018). As a result, WBE is a vital method for establishing and identifying the presence and transmission of both the formerly encountered as well as the newer pathogens infecting humans, like the poliovirus, and the recent SARS-CoV-2, to assess their dissemination, hotspots, and the success of efforts to mitigate these pathogens in a population (Barcelo, 2020). Among the SARS-CoV-2 infection in the communities, 80% of the infected persons are asymptomatic or with minor symptoms (Heneghan et al., 2020). Historically, WBE is used to detect non-enveloped viruses, with polioviruses being one of the most common. Furthermore, wastewater virus titers correspond with the newly reported COVID-19 cases, and wastewater trends are approx. Four to ten days ahead of clinical reports, meaning that wastewater data might be utilized to define public health and hospital planning as an early warning system for potential epidemics. To strengthen COVID-19 response, the CDC (Centre for Disease Control) and other state and local health agencies have initiated wastewater-based monitoring programs, indicating the potential benefit of wastewater surveillance (Mackuľak et al., 2021; Wu et al., 2021). Despite the positive results of prior studies, it is still unknown how effectively wastewater-based epidemiology can detect SARS-CoV-2 genomic variability in a community and in what manner this compares to known viral diversity in the clinical cases. This becomes more crucial as new lineages are discovered. For instance, the B.1.351 strain in the UK has single-nucleotide variants (SNVs) in the spike protein, N501Y (Fontenele et al., 2021), while the B.1.351 strain in South Africa has K417N, E484K, and N501Y (Tegally et al., 2021).

The goal of the present article is to gather and assess information, particularly on different processes of wastewater sampling, its viral load concentration, and methods for the extraction of viral RNA for qualitatively as well as quantitatively detecting SARS-CoV-2 in the wastewater. It aims to emphasize the need for continued effort toward sensitive, specific, cost-effective, wastewater surveillance strategies for emerging and re-emerging pathogens. With the recent surge of JN.1 variant of SARS-CoV-2, this article is very timely to include wastewater surveillance as part of long term strategy for future pandemic preparedness.

### Overview of the studies about detection of SARS-CoV-2 in the wastewater

An overview of study characteristics is presented in Supplementary Table S1. A total of 68 studies from 26 different countries published during 2020 to 2022 reported the detection of SARS-CoV-2 from the wastewater samples. The techniques employed in these studies for concentrating the virus from the wastewater were Polyethylene glycol precipitation technique (24 studies), centrifugation (22 studies), ultrafiltration (13 studies), electronegative membrane filter (4 studies), aluminum-based adsorption precipitation (3 studies), and organic flocculation (2 studies). Out of 68 studies, 66 studies have mentioned the sample size taken for the procedure whereas only two studies were unclear in reporting the sample size. Wastewater treatment plants (59 studies) were the most commonly collected sample sources (Supplementary Table S1). Four studies collected wastewater from water resource recovery facilities (WRRFs), and a single study carried out in Southern Chile, used a sample source of untreated water for the study, whereas eight studies from sewage treatment plants (STPs). Three studies were unclear in reporting the sample type collected. On other hand, regarding genes targeted in these findings, we have found that the N gene (N, N1, N2, N3) was targeted most in 50 research works. Envelope (E), ORF1ab, Spike (S), and RNA-dependent RNA polymerase (RdRp) are some of the other genes that have been exploited as targets. Only three research employed nsp14 primers and probes to identify the membrane (M) gene. Primer sequences listed in Supplementary Table S2 were used by different research groups.

### The likelihood of infection from the SARS-CoV-2 present in the wastewater

The detection of considerable amounts of SARS-CoV-2 in wastewater treatment plants (WWTPs) and subsequent spread of infection has been a matter of concern and learning (Nayak et al., 2020). Since the carriers are assumed to be infected, the transmission of SARS-CoV-2 among human inhabitants may be detected very late and the virus may be discovered only when human testing is available on a massive scale or when the clinical cases are accounted for by health professionals (Figueroa et al., 2017). SARS-CoV-2 is principally respiratory virus and may infect and multiply in the gastrointestinal system (Sun et al., 2020).

COVID-19 patients’ faces, sputum, oro-, and nasopharyngeal swabs, and urine were shown to contain infectious virus particles. This contributes toward entry of viruses into the water systems through a variety of routes, including waste discharged from isolation and quarantine centers of the hospitals, as well as from the houses and other buildings occupied or frequented by infected people, whether symptomatic or asymptomatic (Adelodun et al., 2020; Sun et al., 2020; Xiao et al., 2020). Several studies report that, in addition to the respiratory symptoms, a proportion of SARS-CoV-2 infected patients exhibits diarrhea as well. Infection of SARS-CoV-2 to the gastric or rectal epithelial cells ultimately releases infecting virion particles to the entire gastrointestinal tract (Song et al., 2020). Thus, the excreta of SARS-CoV-2 infected patient may contain a huge number of viruses that can be released into the municipal sewage system and remains active for a long time, putting operators at risk of infection. Wastewater is also suspected of being at least partially responsible for a prior SARS-CoV-2 outbreak due to inadequate ventilation.

Even though the bulk of studies have demonstrated that variations in viral infection levels in sewage are not driven by the predictable principles throughout time. According to various studies, virus infection levels in sewage change steadily with considerable seasonal oscillations (Abe et al., 2016; Kitajima et al., 2020). It is identified that diurnal patterns during sample periods, have not demonstrated any notable changes in the viral titers. To summarize, according to the World Health Organization (WHO), from neither untreated nor treated sewage SARS-CoV-2 infection has been spread. In the municipalities of some countries, the components of the SARS-CoV-2 RNA have been found in untreated sewage and sludge. It is not always the case that the presence of viral genetic material (RNA) indicates the existence of a live infectious virus or an infection risk. RNA fragments often last considerably longer in the sewage matrices than an active virus, and hence there is typically no strong relation between viral load and infectiousness in the sewage samples (Amereh et al., 2021) which have a short life cycle when they are not within a host cell, unlike other microorganisms (such as bacteria). SARS-CoV-2 was also inactivated in the aqueous environment as a result of lipid envelope degradation; however, the process is not as rapid as it could have been. The presence of SARS-CoV-2 in municipal wastewater was investigated in the Netherlands (Medema et al., 2020). According to different studies, there is no published research on the stability and survival of SARS-CoV-2 in the wastewater in terms of viral vitality (Tran et al., 2021; Amahmid et al., 2022). Most studies for detecting and quantifying the virus in sewage did not include viral viability. According to research done on culture cells (Rimoldi et al., 2020) the virus’s infectivity in wastewater was zero (Wang et al., 2020). Viral outgrowth technique was used to assess SARS-CoV-2 infectivity in raw wastewater and found no infectivity (Westhaus et al., 2021).

### Outgrowth of SARS-CoV-2 in the wastewater

Hand washing, sputum, vomit, and primary drainage outlets that flow straight into river bodies are some of the sources where coronavirus can enter wastewater (residential outlets, hospitals, and river bodies). It is also reported that SARS-CoV-2 infection-related viral shedding is found in human urine. However, the primary method that has received the most attention is the release of viral RNA in the feces of infected people. The SARS-CoV-2 virus can be detected in the feces of either asymptomatic or symptomatic individuals. Even after getting negative SARS CoV-2 results through throat swabs and urine sample testing, few studies have reported that the viral RNA can be present for up to 10 days in the fecal samples. The sewage systems invariably contain human feces, which might create the perfect environment for the enteric virus to reproduce and spread via aerosols carrying the virus. It is also a significant source of disease transmission because of this unrestrained discharge of wastewater into the environment and the movement of microbial pollutants to people and other living things. Since it offers effective, thorough, and real-time monitoring data, WBE analysis of population-pooled wastewater is a desirable choice for public health monitoring. WBE is currently recognized as a practical technique for early monitoring and detecting the enveloped SARS-CoV-2 virus as in Spain, where the virus was seen to be present 41 days earlier than the first recorded COVID-19 case. The deployment of effective lockdown measures as a result of wastewater surveillance in Spain allowed for earlier detection of the second wave, which helped to lessen the pandemic scenario. The second wave of the viral epidemic in Hungary was likewise accurately predicted by wastewater surveillance, demonstrating the value and affordability of this method for outbreak identification (Róka et al., 2022). The amount of viral genetic material shed per gram of infected feces may be the key determinant of the presence of coronaviruses in the wastewater. However, further research is needed to determine how often there is an excretion of coronaviruses in the feces and urine of diseased people. These details may be crucial in assessing the possibility of fecal transfer because it will not only provide an indication of the concentrations predicted in wastewater. The correlation of the viral load in the wastewater with the rate of infection in the population is made easier with the knowledge of frequency of shedding.

### Wastewater detection techniques for SARS-CoV-2

Wastewater-based viral detection has been the focus of several studies. The amount of the sample, yield of the nucleic acid extracted using nucleic acid-based processes, and purity, have an impact on the accuracy of virus detection. According to reports from across the world, numerous human viruses have been studied and found in wastewater systems in the WWTPs at various sample locations. Several methods have been used for the identification as well as quantifying the viruses in the wastewater systems *viz.* pulsed-field gel electrophoresis, epifluorescence microscopy, immunofluorescence testing, electronic transmission microscopy, conventional cell culture, flow cytometry, and molecular techniques (Corpuz et al., 2020). These methodologies largely yield independent information on the existence of viruses in sludge and wastewater samples (qualitative and quantitative data). Most of the molecular techniques for measuring the size of a virus rely on counting the number of genetic material segments present in the virus. Even if the virus is inactivated, i.e., if the viral capsid or envelope has been destroyed, or if the viral genetic information is incoherent, the molecular techniques can identify. In contrast, cell culture-based and immunological techniques are commonly used to determine viral vitality (Ali et al., 2021). The present study has focused on six different detection techniques used for SARS CoV-2.

Due to the lack of well validated protocols and methods, WBE monitoring of SARS-CoV-2 has been a challenging task. Wastewater sampling, viral concentration RNA extraction, detection, and data processing are the major steps of the protocol followed for WBE (Kumblathan et al., 2022). Most of the research used culturable viruses and or bacteriophages as model viruses to develop concentration methods for different non-enveloped enteric viruses like enterovirus, norovirus, hepatitis A virus, and adenovirus. Numerous techniques for concentrating viruses in wastewater have been developed over a period of time. Enteric viruses have been concentrated using electropositive or electronegative membranes in untreated and treated wastewater samples (Sherchan et al., 2020).

**Figure 2:**
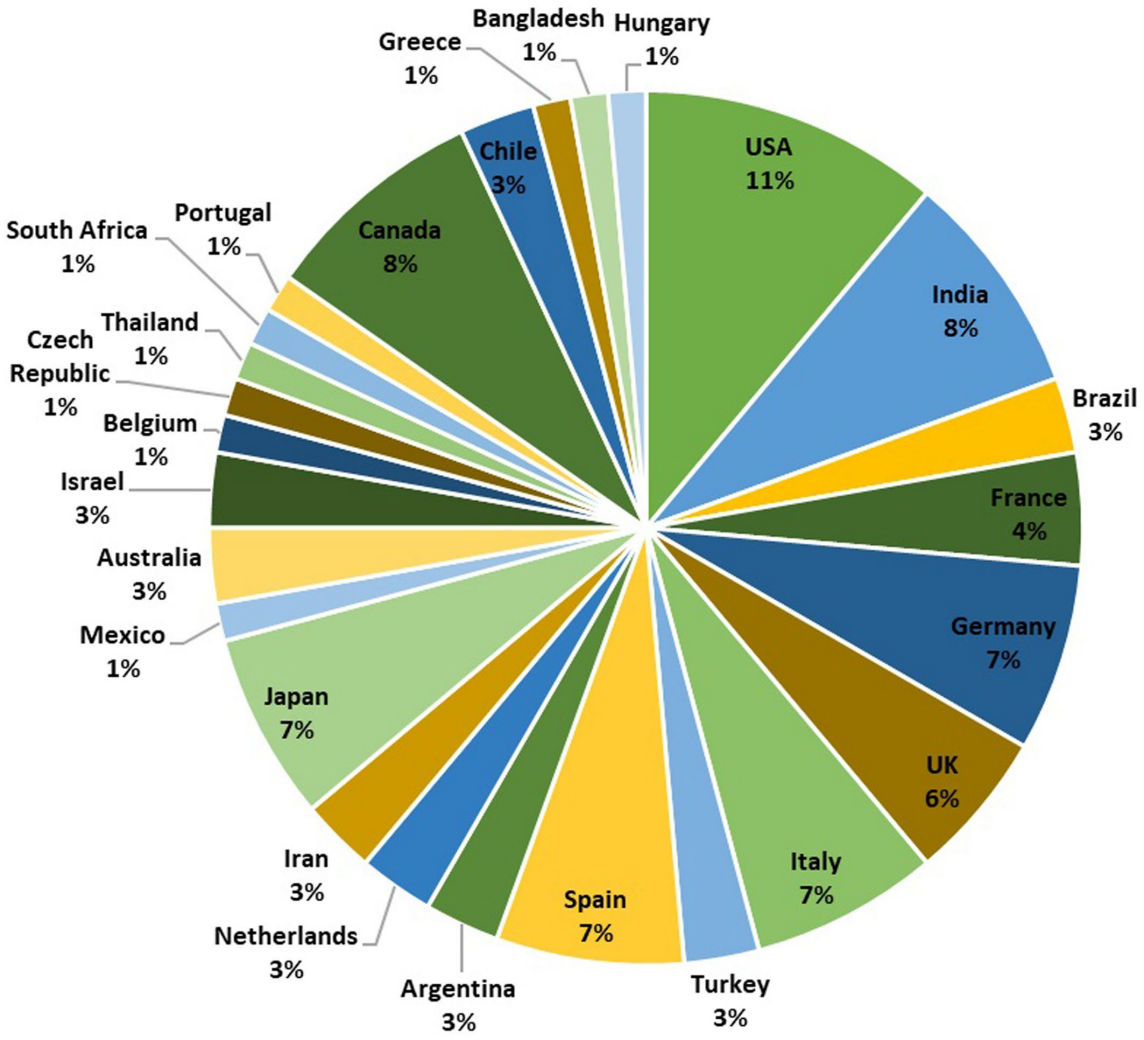
A pie chart representing the global distribution of SARS-CoV-2 wastewater-based epidemiology surveillance studies conducted in 26 different countries. The chart is based on a total of 72 studies. The number of studies conducted in each country is as follows: USA (8), India (6), Brazil (2), France (3), Germany (5), UK (4), Italy (5), Turkey (2), Spain (5), Argentina (2), Netherlands (2), Iran (2), Japan (5), Mexico (1), Australia (2), Israel (2), Belgium (1), Czech Republic (1), Thailand (1), South Africa (1), Portugal (1), Canada (6), Chile (2), Greece (1), Bangladesh (1), and Hungary (1). This pie chart provides an overview of the distribution of SARS-CoV-2 surveillance efforts in various countries, reflecting the global importance of wastewater-based monitoring.

Discharges from various parts of the hospital, research laboratory, laundry, contain an extensive variety of macro and micro-pollutants, along with a variety of bacteria and viruses like SARS-CoV-2. The prevalence and survival of the SARS-CoV-2 virus in water, wastewater, and rivers are now being investigated in several research (Carducci et al., 2020; Guerrero-Latorre et al., 2020; Naddeo and Liu, 2020; Rimoldi et al., 2020; Vogels et al., 2020). Detecting SARS-CoV-2 in wastewater in several places like hospital effluent, sewage, using an array of methods has been reported. The detection approach is preceded by various stages of virus concentration such as Ultrafiltration, PEG precipitation, and electronegative membrane adsorption, after which viral RNA extraction is done directly. Followed by RNA extraction, RT-qPCR or (nested) RT-PCR have been widely employed to detect the SARS-CoV-2 RNA (Ahmed et al., 2020; Haramoto et al., 2020; Lodder et al., 2020; Medema et al., 2020; Nemudryi et al., 2020; Randazzo et al., 2020; Rosa et al., 2020; Wang et al., 2020; Wu et al., 2020; Wurtzer et al., 2020). It is reported that SARS-CoV-2 RNA was successfully identified in wastewater and hospital wastewater using various viral concentration methods in countries like France, Netherlands, Spain, Italy, United States, and Australia (Achak et al., 2021). The SARS-CoV-2 detection accomplished with the objective of WBE surveillance in different country globally are depicted in Figure 2.

**Figure 3:**
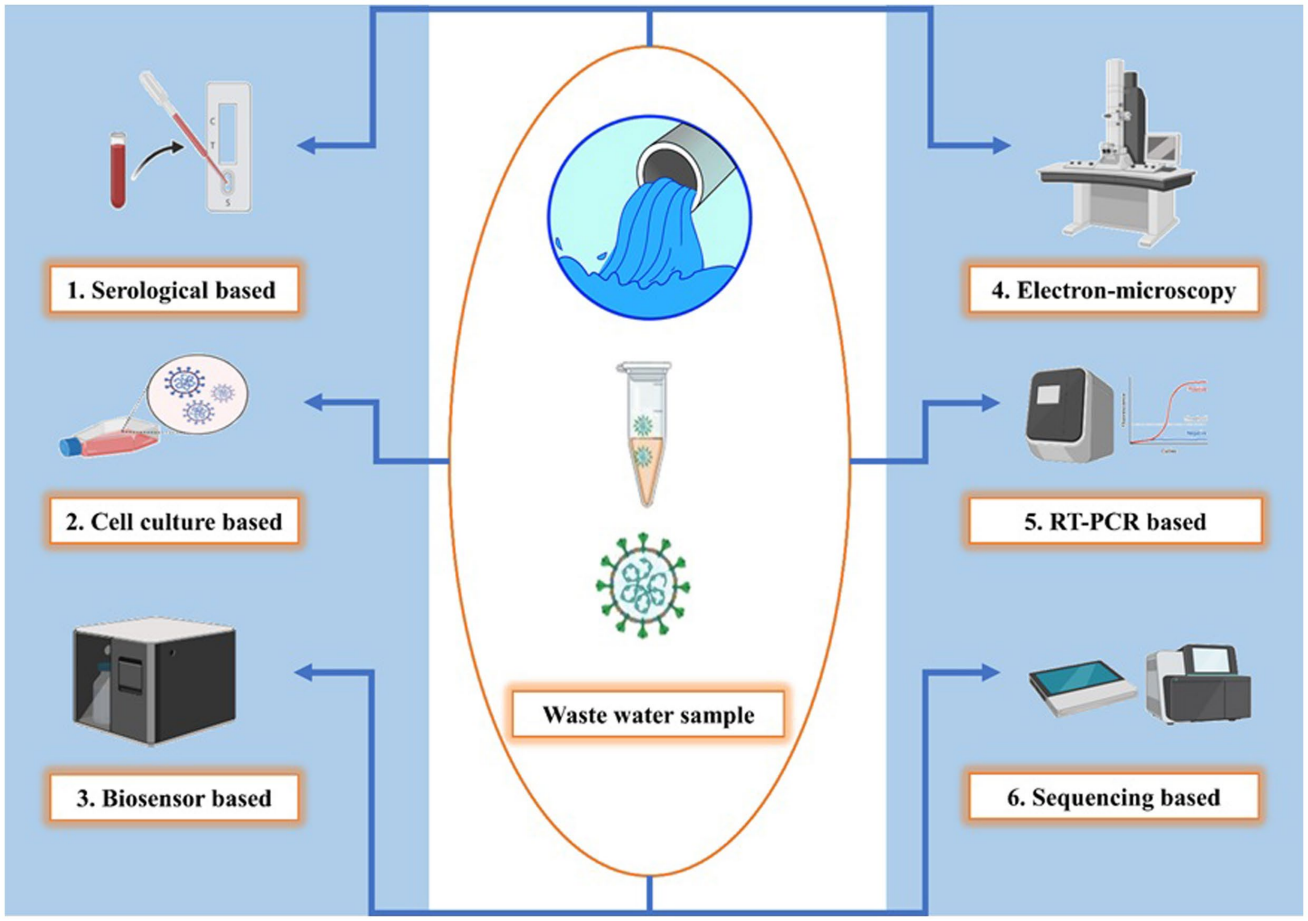
Diverse SARS-CoV-2 detection techniques in wastewater. Serological-based detection uses ELISA and Western blotting to identify antibodies, Cell culture reveals infection’s cytopathic effects, Biosensors capture viral particles with immobilized antibodies, Electron microscopy directly visualizes virions using TEM, RT-PCR amplifies and quantifies viral RNA, Sequencing identifies genetic sequences in the wastewater metagenome and this figure showcases the versatility of SARS-CoV-2 monitoring methods in wastewater, each with its unique approach to detection and quantification.

Detecting and monitoring SARS-CoV-2 in wastewater are presently divided into three categories: (1) qualitative molecular technique, (2) quantitative molecular approaches, and (3) *in vitro* plaque-forming unit counts (PFU). The RNA of SARS-CoV-2 is the focus of molecular methods, which can quantify the number of RNA copies (or fragments) in a water sample but not viral infectivity (Johnston and Behrens, 2020). PFU can offer a quantitative estimate of infective virions, however, this method is time-consuming and difficult to implement because *in vitro* production necessitates the use of a suitable host (Greening et al., 2002). It should be noted that the cytotoxicity of toxins commonly found in wastewater samples can reduce the sensitivity of plaque assays for virus identification. In addition, compared to RNA detection, viral concentrations must be significantly greater to isolate infective virions (>106 copies ml^−1^). Therefore, it is not surprising that the wastewater from hospitals tested positive for SARS-CoV-2 RNA, but not for infective virions. However, due to the extensive use of disinfectants and different surfactants in hospital wastewater, the concentration of infective virions may have been below the detection limit. SARS-CoV-2 detection and enumeration in wastewater are particularly difficult, regardless of the methods utilized, due to their low abundance after dilution (101 to 106 copies ml^−1^) compared to direct assays on human excretions. As a result, a high-recovery rate sample concentration is required, and promising approaches are needed (Bogler et al., 2020). Following techniques (Figure 3) could be employed for the detection of SARS-CoV-2.

#### Serological detection

Some immunological detection methods based on a particular antibody to the target antigen have been developed, but it may be challenging to use these with environmental samples because it requires a high viral load in the sample, around 10,000 to 100,000 viral particles per milliliter. A quick and simple method for measuring viral protein or detecting the entire virus is the enzyme-linked immunosorbent assay (ELISA) (Engvall and Perlmann, 1971; Crowther, 2009). After binding with a particular antibody, the target antigen is recognized by a colorimetric chromatic response in a variety of antigen detection ELISAs.

#### Biosensor detection

A wide range of chemicals may be analyzed using biosensors. Viruses are one of several analytes that may be detected and measured using different biosensors. By delivering sensitive, quick, low-cost, selective, and portable devices, viral biosensors have the potential to replace current diagnostic approaches. Typically, sensors consist of two parts: (i) a transducer for the transformation of the recognition procedure into a signal (optical, electrochemical, acoustic, or calorimetric) which can be treated for further quantification; and (ii) biochemical or biological recognition layers, which are one type of receptors that interact with a particular analyte (Mustafa and Andreescu, 2018).

#### Electron microscopy detection

Transmission electron microscopy (TEM) may Be used To identify intact virus particles, just As It does with other viruses. This approach Has morphological localization In certain cells and subcellular compartments As an advantage. Another benefit Is The ability To detect whole virus particles rather than structural components such As proteins and RNA, which may Be detected using other approaches. As TEM Is highly selective To host-specific infectious virus, depicting Its drawback In using for The detection of virus for WBE. Additionally, investigation of large number of samples for The detection of viruses using TEM Is difficult As It requires sophisticated equipment and The expertise of trained technician.

#### Next generation sequencing technique

High-throughput next generation sequencing of metagenome from wastewater or sludge has been a recent development in the monitoring of wastewater for microbiological and viral contamination (Johnston and Behrens, 2020). The procedure entails collecting huge datasets, reading single-nucleotide signals sequentially for each of the nucleic acid fragments present in the sample, combining them using bioinformatic tools, and comparing the results to an open-access database containing reference genome sequence (Liu et al., 2012).

#### RT-PCR detection

Different RT-qPCR kits are commercially available, with a few of them having previously received COVID-19 certification for regular COVID-19 diagnostic testing (Lowe et al., 2020; van Kasteren et al., 2020). It has evaluated the analytical effectiveness and sensitivity of four RT-qPCR techniques especially for assessing the performance of primer-probe sets (Vogels et al., 2020). The findings indicated that, while all primer-probe sets were efficient in detecting SARS-CoV-2, there were considerable differences in analytical sensitivity in circumstances when the viral load was very low (Vogels et al., 2020). These kits, which have been widely accepted in clinical science, might likewise be utilized in WBE.

#### Cell culture-based detection

Cell culture-based methods such as the “plaque test” and “tissue culture infectious dose-50” (TCID50) was used to detect infectious viruses in the environmental pollutants (Mattison and Bidawid, 2009; Calgua et al., 2011). According to the study, the immunofluorescence test, which combines cell cultures with antibody detection, is based on the presence of a viral protein in the host cell interacting with a specific antibody. Specific fluorescent microscope makes it possible to see the fluorescent signal in various cell sections, for example, cell membrane, nucleus, and cytoplasm. This fluorescent signal is produced by the identified antibody that has been with a fluorescent-labeled dye (fluorescein isothiocyanate) (Dilnessa and Zeleke, 2017).

Cell culture and PCR techniques are used to identify and quantify viruses. The manor benefit of using this method is that it enhances sensitivity and detection speed while overcoming both systems limitations and combining their strengths. Although all viruses cannot result in cytopathic effects and plaques as well as some of the viruses cannot be grown *in vitro* conditions, the procedures are time-consuming and expensive (Greening et al., 2002; Haramoto et al., 2018).

### Overall process for WBE of SARS-CoV-2

Global urbanization, along with fast population expansion and the frequent introduction of hazardous viral components to the human population, necessitates the expansion of quick surveillance systems to track viral dissemination and infection status (Mao et al., 2020). Most essentially, this testing strategy must continue to expand test coverage (i.e., the proportion of the population examined) until a critical threshold is reached. This level refers to the number of tests required to indicate a single positive (confirmed) case.

#### Sample collection and transportation

High-frequency automated samplers were used to collect wastewater samples, composite samples of 24 h of untreated wastewater from different sites as head works of the wastewater treatment plant, medical institutions, and wastewater collecting systems. Wastewater samples from several locations with a variety of compositions, as measured by carbonaceous biochemical oxygen demand (CBOD), total suspended solids (TSS), and ammonia (NH4-N), were collected in order to evaluate the durability of the various concentration techniques (LaTurner et al., 2021). Most samplers contained refrigeration or were supplied with ice/dry ice in order to maintain the inside collecting vessel’s temperature. The approach previously described was used to process samples within 1–3 days of receipt (Wu et al., 2021). To summarize, the UV light was employed to sterilize the outside of the sample’s container (20 min) before handling, and pasteurization (heating at 60°C in a water bath for 90 min) was utilized to inactivate microorganisms in sewage. To eliminate cell debris and solid components, pasteurized samples will be vacuum filtered using a 0.22-m polyether-sulfone membrane. The virus particles were concentrated in the supernatant as indicated below, and the rest were be kept at 4°C (Wu et al., 2021). Three aliquots of 500 mL each will be taken from the composite sample. The wastewater collection did not require any permits (Nemudryi et al., 2020). The samples from the other utilities were transported overnight on ice and processed the next day. The samples can be kept at 4°C until they were processed further. Samples that were delayed in shipping or exposed to extreme temperatures (>10°C) will not be processed (Ai et al., 2021; Gregory et al., 2021).

#### Sample processing and extraction

A 0.45 μm and a 0.2 μm polyethersulfone (PES) filter was utilized for each aliquot of the composite wastewater sample. Utilizing Amicon^®^ Ultra 15 Centrifugal Filter Units (Millipore, Sigma, United States), total wastewater aliquot is concentrated. The procedure was carried out five times for every sample using two filter units, and then the concentrates were pooled for each sample (from the two filter units). Using the RNeasy mini kit (Qiagen, United States), total RNA was extracted from a 200ul concentrated wastewater aliquot of each sample (Nemudryi et al., 2020; Ai et al., 2021; Gregory et al., 2021).

### Quantitative real-time PCR for detection of SARS-CoV-2 and quantification of viral load

SARS-CoV-2 viral RNA was detected using approved RT-PCR kits in the respective countries. For, e.g., in India, Indian Council of Medical Research (ICMR) recommendations was taken into account for the same. The reactions were performed on an Applied Biosystems QuantStudioTM 5 Dx Real-Time PCR equipment and analyzed as directed in the manual. The viral load of RT-PCR positive samples were determined using a two-step quantitative RT-PCR procedure with absolute quantification, and the viral particle copy number was represented as normalized copies per volume of the sample.

### Concentration techniques for the SARS-CoV-2 from wastewater

#### Ultrafiltration

Ultrafiltration is widely used for concentrating wastewater to obtain viruses. In UF applications, small amounts of centrifuged or filtrated wastewater samples of 40 to 400 mL were used, with Relative Centrifugal Force (RCF) in the range of 3,000–4,000 g. In this procedure, centrifugal filters were used with nominal molecular weight limits (NMWL) varying from 10 to 150 kDa (Mousazadeh et al., 2021). Reportedly, UF, specifically double UF, gave a more accurate outcome than the other concentration methods studied. Therefore, it might be adopted as a protocol due to its uniformity, adequate recovery rate, absence of eluting, and exceptionally short operating time (max 1 h) (Cervantes-Aviles et al., 2021). Ultrafiltration for viral extraction, unlike PEG-based separation and electropositive membrane filtration, does not require the preconditioning of water samples, allowing its application in a wide range of water quality circumstances (Hill et al., 2007). Multiple layers of asymmetric cellulose acetate membranes were used to separate flow channels for raw water samples and driving solution in the first ultrafiltration research to concentrate poliovirus from a water sample, achieving a recovery efficiency of 95–100% from a huge volume of water of 10 L (Sweet et al., 1971).

#### Polyethylene glycol precipitation

Precipitation by polyethylene glycol (PEG) is a two-phase partition of aqueous polymers based on the liquid–liquid partition (Ikner et al., 2012) wherein PEG enhanced precipitation to concentrate SARS-CoV-2 genetic material in the wastewater (Wu et al., 2020). According to a method, the first stage for inactivating the virus is pasteurization at 60°C for 90 min in a water bath. For the removal of bacterial particles and other debris, filtration of pasteurized water samples could be done using a 0.22-micron membrane. The concentration of the viral load is carried out by precipitation of the filtrate with PEG 8000 and NaCl. The presence of SARS-CoV-2 viral RNA in raw wastewater samples from the Old Pirana WWTP in Ahmedabad, Gujarat, India, was discovered using PEG precipitation (Kumar et al., 2020).

#### Virus adsorption-elution

Virus adsorption-elution (VIRADEL) concentrates viruses from wastewater using electronegative or electropositive membrane filters. The attachment of virus particles to a cellulose nitrate membrane filter with a pore size of 0.45 microns is accomplished by salt bridging, which is occasionally aided by the injection of MgCl_2_ or NaCl during electronegative membrane filtration (ENMF) (Ikner et al., 2012). ENMF was used for concentrating the unprocessed wastewater with and without several pre-conditioning treatments, including acidified (2 N HCl; pH 4) and MgCl_2_-supported (a final concentration of 25 mM) (Ahmed et al., 2020). Using murine hepatitis virus (MHV), the RNA recovery performance of these options was compared to CeUF, PEG 8000 precipitation, and ultracentrifugation. Based on the limited data available, it was determined that preconditioning procedures such as adding MgCl_2_ to ENMF might efficiently increase SARS-CoV-2 recovery, as well as electropositive filters, could concentrate the SARS-CoV-2 virus from wastewater. As a result, extensive study is required to evaluate and enhance the efficacy of both concentration procedures (Kabdaşlı and Tünay, 2021).

#### Skimmed milk flocculation and aluminum-driven flocculation

Viruses isolated from wastewater with glycine alkaline buffer are flocculated with skimmed milk at pH 3.5 during the skimmed milk flocculation (SMF) technique which improves the wastewater concentration (Calgua et al., 2013). At pH 6.0, viruses are adsorbed on new Al (OH)_3_ flocs produced by aluminum-driven flocculation. This concentration method has only been used to extract SARS-CoV-2 genetic material from wastewater (Randazzo et al., 2020). The first research utilized samples from three WWTPs in Valencia, Italy (Randazzo et al., 2020), whereas the second research used samples from six WWTPs in Murcia (Spain), which is relatively low-frequency site in the nation but a step in the right direction. The validation of this technique was done with the Porcine Epidemic Diarrhea Virus (PEDV) and Mengovirus (MgV). Skimmed milk flocculation, aluminum-driven flocculation for wastewater, and polluted receiving water might be suitable substitutes provided additional research confirms their excellent performance, perhaps with minor tweaks.

### SARS-CoV-2 removal from wastewater treatment facilities

**Figure 4:**
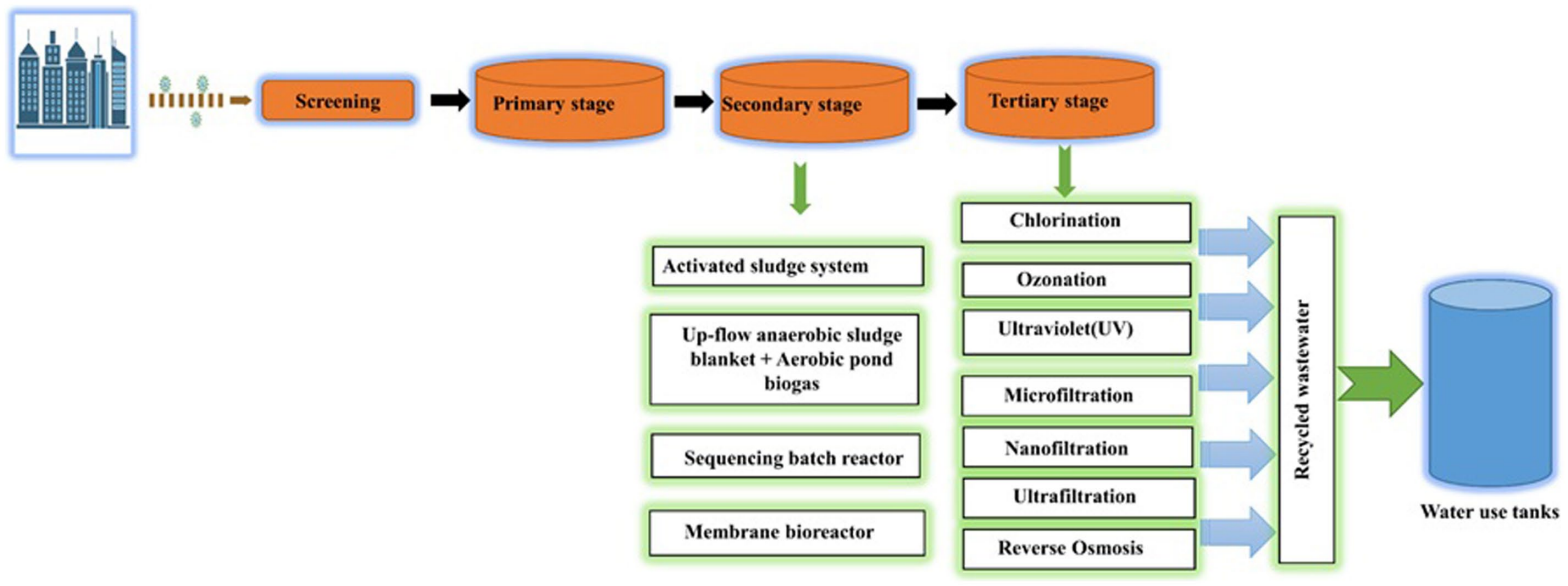
Schematic representation of a WWTP (Waste Water Treatment Plant) process. Key stages include: Screening: Initial removal of large debris for equipment protection, Primary Stage: Sedimentation and flotation to separate solids and organic matter, Secondary Stage: Microbial breakdown of organics for further purification, Tertiary Stage: Advanced treatment with coagulation, filtration, and disinfection for high-quality effluent, Recycled Wastewater: Treated water for non-potable use, resource conservation, Water Use Tanks: Storage for non-potable applications, promoting water sustainability.

In order to prevent the downward spread of SARS-CoV-2, wastewater treatment facilities must be disinfected for inactivation and eradication. For the SARS-CoV-2 wastewater treatment facilities, treatment like primary, secondary, and tertiary processing stages may be taken into consideration. The first stage of treating wastewater for the removal of SARS-CoV-2 is the elimination and inactivation of fixed and volatile suspended solids (VSS) present in the wastewater through physical barriers (Saawarn and Hait, 2021). As the first step in the treatment of wastewater, flocculent precipitation, adsorption, and gravity precipitation are used. It is hard to completely eradicate SARS-CoV-2 from wastewater during the initial stage. On the other hand, the second stage depends on the biological treatment process to eliminate biodegradable organic compounds through the cellular activity of microbes (Liu et al., 2020). A membrane bioreactor (MBR), sequencing batch reactor (SBR), moving bed biofilm reactor (MBBR), pond system, up-flow anaerobic sludge blanket (UASB), activated sludge process (ASP), and membrane treatment is used to facilitate this step of treatment. The final step of wastewater treatment in WWTPs before discharging treated water of higher quality into the environment is referred to as the tertiary stage and comprises procedures like chlorination, UV disinfection, ozonation, membrane technology (Gerba and Pepper, 2019; Saawarn and Hait, 2021). This step of the process focuses on the emission quality as well as the underlying chemical or physical processes. The steps of wastewater treatment in WWTPs are shown in Figure 4.

### Summary and future perspectives

Because some of them have little or no access to clean and adequately treated water, low-income groups are one of the most affected by this latest outbreak. Investigations into the dangers of SARS-CoV-2 virus transmission through feces are critical, especially in areas where sanitation systems are not well-managed (Amahmid et al., 2022). Under epidemiologically sensible limits, conditional applications of detecting SARS-CoV-2 viral load in wastewater is a progress in the right direction toward development of novel techniques (Galani et al., 2022). Although the analytical techniques of SARS-COV-2 WBE has the several limits and hurdles to overcome (Alygizakis et al., 2021), it is potent and cost-effective approach for determining a population’s lifestyle exposure and health condition. This method helps in decision-making for authorities to quarantine, intervene, and lockdown (targeted or broader) to stop the outbreak in a certain area as it gives real-time data and can be used as an early warning system. It has been stated that viral transmission through wastewater systems has yet not been confirmed for the current scenario of the SARS-CoV-2 pandemic due to a lack of enough data for COVID-19 transmission by wastewater exposure. It is also interesting to note that, most of the disease-causing pathogen or microorganisms has been removed during processing of wastewater in the wastewater treatment plants. As a result, compared to human-to-human interaction, virus exposure to human through wastewater is deemed low.
Even though very few studies have been found for understanding the survivability of the virus in wastewater, most of them only focused on the *in vitro* study of the virus using a viral surrogate. As a result, the survival rates of the SARS-CoV-2 virus differ from wastewater and inter-human transmission (Amoah et al., 2020). COVID-19 data in wastewater does not offer reliable data on infection rates when compared to clinical data that provides infection rates based on live viruses since the current technology analyses the total viral particle regardless of its active or inactive state. Although a few research has looked at coronavirus survival in wastewater under outdoor circumstances, they have all been laboratory-based studies employing viral surrogates or pasteurized wastewater. As a result, in field circumstances, the survival rates may change (Amoah et al., 2020). The lack of attention might be attributable to the former idea that these viruses may not exist in wastewater or that they will be found in low viral levels, if they do. However, mounting data suggests that this is not the case, necessitating research into how traditional wastewater treatment procedures may either remove or inactivate coronaviruses (Amoah et al., 2020).
Before symptoms manifest, the uncontrolled COVID-19 mutation may be discovered by utilizing WBE through continuous wastewater monitoring. This would be a key strategy for pre-emptive detection of the viral evolution, manifested through different variants of SARS-CoV-2 (for example). Looking in future, we may be better placed to detect the emerging variants, as was in the case of SARS-Cov-2 variants of concern – Alpha, Delta, Omicron, Recombinants and now JN.1. The same would be true for other viral infections like Dengue which is a yearly disease of concern for countries with tropical climate in the south Asia. As a result, wastewater monitoring can help forecast an impending problem throughout the world, especially if clinical surveillance capacity is limited. WBE data give an early indicator of human health that may be utilized to more intelligently diagnose and manage infectious disease transmission in the future (Pulicharla et al., 2021). WBE can give additional information on illness patterns in a community, which will become more significant as clinical testing rates drop. However, achieving an accurate evaluation of disease occurrence (total case numbers) using WBE alone would include numerous unknowns at a local level that would be difficult to collect without extensive, resource-intensive local research, which would negate the cost-effectiveness of WBS otherwise (Hrudey and Conant, 2022).
There are no quantitative data on infectiousness and the chance of transmission from fecal contamination. While SARS-CoV-2 RNA has been found in sewage and wastewater, no indication of infectiousness has been found in these sources, and a transmission risk from these sources is judged improbable based on the research reviewed. Quantitative data on infectious viruses, and information on the anticipated infectious dosage in humans, are required to appropriately assess these hazards (Heneghan et al., 2021). Furthermore, in-depth NGS analysis of wastewater samples might enhance public health decision-making by detecting changes in viral diversity, which can suggest the introduction of epidemiologically or clinically significant alterations (Izquierdo-Lara et al., 2021). A precise and informative model that uses WBE-derived data may be developed using informed knowledge of viral behavior in the micro-environment. Such a model might aid in the development of technology tools and practical strategies to alleviate the public health and economic impacts of ongoing viral epidemics or other prospective outbreaks. The lessons learned from the worldwide experience include that strong contract tracing and containment strategies are required to keep the disease under control until an authorized treatment is available.

## Author contributions

GG: Formal analysis, Investigation, Methodology, Resources, Visualization, Writing – original draft. SS: Resources, Writing – original draft. EK: Data curation, Writing – original draft. DK: Resources, Writing – original draft. PG: Data curation, Writing – original draft. SK: Resources, Writing – original draft. HM: Resources, Writing – original draft. MI: Visualization, Writing – review & editing. RP: Project administration, Supervision, Visualization, Writing – original draft. PB: Conceptualization, Funding acquisition, Supervision, Visualization, Writing – review & editing.

## Footnotes

^1^ https://www.who.int/emergencies/disease-outbreak-news/item/2020-DON229
