# Supplementary material for: An interpretative review of the wastewater-based surveillance of the SARS-CoV-2: where do we stand on its presence and concern?
Source: Front Microbiol. 2024 Jan 22;15:1338100. doi: 10.3389/fmicb.2024.1338100 (PMC10839012; doi:10.3389/fmicb.2024.1338100)
Supplement: Supplementary file 1 [file Table_1.DOCX]

**Supplementary information**

**An interpretative review of the wastewater-based surveillance of the SARS-CoV-2: where do we stand on its presence and concern?**

Gayatri Gogoi^1,4^, Sarangthem Dinamani Singh[^1^](#a), Emon Kalyan^1^, Devpratim Koch^1,4^, Pronami Gogoi[^1^](#a), Mrinmoy Kshattry[^1^](#a), Hridoy Jyoti Mahanta^2,4^, Md Imran^3^, Rajesh Pandey^3,4, *^, Pankaj Bharali^1,4, *^

^1^Center for Infectious Diseases, Biological Science and Technology Division, CSIR-North East Institute of Science and Technology (CSIR-NEIST), Jorhat-785006, Assam, India.

^2^Advanced Computation and Data Sciences Division CSIR-North East Institute of Science and Technology Jorhat 785006 Assam India

^3^Division of Immunology and Infectious Disease Biology, INtegrative GENomics of HOst-PathogEn (INGEN-HOPE) laboratory, CSIR-Institute of Genomics and Integrative Biology (CSIR-IGIB), Mall Road, Delhi-110007, India.

^4^Academy of Scientific and Innovative Research (AcSIR), Ghaziabad-201002, India.

*Co-corresponding authors

*Emails for correspondence: [rajeshp@igib.in](mailto:rajeshp@igib.in) / [pbharali@neist.res.in](mailto:pbharali@neist.res.in)

**Contact Details**

**Rajesh Pandey, PhD**

Principal Scientist,

INtegrative GENomics of HOst-PathogEn (INGEN-HOPE) laboratory,

CSIR-Institute of Genomics and Integrative Biology (CSIR-IGIB),

North Campus, Near Jubilee Hall, Mall Road, Delhi-110007, India.

Email: rajeshp@igib.in; rajesh.p@igib.res.in; Phone: 011-27002200 (Ext. 254)

**Table S1.** Detection of SARS-cov-2 in wastewater

**Table S2.** Primers/Probes used for the amplification of SARS-CoV-2 RNA in wastewater

**Table S1: Detection of SARS-cov-2 in wastewater**

| Sl. No. | Study | Location | Sample source | Volume used (ml) | Removal of large debris and suspended solids | Concentration method | Qpcr assay |
| --- | --- | --- | --- | --- | --- | --- | --- |
|  | [[1](#Fonteneleetal2021)] | USA | The wastewater treatment plant, hospital facilities | 150 ml | Polyethersulfone (PES) filtration | Amicon® Ultra 15 Centrifugal Filter U | SARS-cov-2 detection assay targeting the E gene |
|  | [[2](#Wuetal2020)] | USA | Wastewater treatment plants or catchments in 40 U.S. states | 40-ml | Vacuum filtration with 0.22-μm polyether sulfone membrane | Centrifugation | Taqman® Fast Advanced Master Mix and U.S. CDC N1, N2 primer/probes. |
|  | [[3](#Nemudryietal2020)] | Bozeman, Montana (USA | Municipal wastewater treatment plant | - | Filtration | Ultrafiltration | N1 and N2 |
|  | [[4](#LaTurneretal2021)] | USA | Wastewater treatment plants (wwtps) | 50 ml | Centrifugation | The Amicon® Pro Purification System | N1 SARS-cov-2, N2 SARS-cov-2 |
|  | [[5](#Aietal2021)] | USA | Wastewater treatment plants (wwtps | 100 ml | Filtration | Organic flocculation and centrifugal ultrafiltration | (N) gene regions and the envelope (E) gene of SARS-cov-2 |
|  | [[6](#Raineyetal2022)] | USA | WWTP | 50 ml | Electronegative membrane filtration | Centrifugation | RT-PCR assays that target regions of the ncov nucleocapsid gene |
|  | [[7](#Fonteneleetal2021)] | USA | Wastewater treatment plant | 150 ml | Polyethersulfone (PES) filtration | Amicon 174 ® Ultra 15 Centrifugation | E gene |
|  | [[8](#Gregoryetal2021)] | USA | WWTF | 37.5 ml | Polyethersulfone membrane filtration | Centrifugation | - |
|  | [[9](#Kumaretal2021)] | INDIA | WWTP | 30 ml | Filtration | Centrifugation | ORF1ab, N |
|  | [[10](#Kumaretal2020)] | Ahmedabad, Gujarat, India | WWTP | 50 ml | Filtration | Centrifugation | ORF1ab, N gene and S gene |
|  | [[11](#Dharmadhietal2021)] | Pune, India | WWTP | 200 ml | Filtration | Centrifugation | TRUPCR® SARS-cov-2 RT qpcr kit (V-3.2) |
|  | [[12](#Kumaretal2021)] | India | Treatment plants | 30 ml | Filtration | Centrifugation | ORF1ab, N Protein, and S Protein |
|  | [[13](#Kumaretal2021)] | Ahmedabad, India | Wastewater pumping stations and sewage treatment plant | 30 ml | Filtration | Centrifugation | ORF1ab, N Protein, and S Protein |
|  | [[14](#Claroetal2021)] | Sao Paulo, Brazil | WWTP | 40 ml | Centrifugation | Centrifugation | N1 and N2 gene assay |
|  | [[15](#Fongaroetal2019)] | Florianopolis, Santa Catarina, Brazil | Sewage system | 200 ml | - | Polyethylene glycol precipitation | Rdrp, S and N regions |
|  | [[16](#Wurtzeretal2020)] | Paris, France | WWTP | 100 ml | - | Centrifugation | E gene by RT-qpcr |
|  | [[17](#Wurtzeretal2021)] | Paris, France | WWTP | 200 ml | - | Centrifugation | Rdrp and E genes |
|  | [[18](#Agarwaletal2021)] | Germany | WWTP | 1000ml | - | Electronegative membrane filter | N, S, and ORF1ab |
|  | [[19](#Rubioetal2021)] | Germany | Sewage system | 50 ml | Ultracentrifugation | Centrifugation | N1 |
|  | [[20](#Dumkeetal2021)] | South-east Germany | WWTP | 40 ml | Centrifugation | PEG (MW 8000)/nacl precipitation | E, and S gene |
|  | [[21](#Fitzgeraldetal2021)] | UK | WWTP | 40 ml | Filtration | Centrifugation | E and N1 |
|  | [[22](#Hillaryetal2021)] | UK | WWTP | 50–100 ml | Centrifugation | Ultrafiltration | E and N1 |
|  | [[23](#Martinetal2020)] | South East England, UK | Sewage plant | 500 ml | Filtration | Centrifugation | Rdrp and E-Sarbeco |
|  | [[24](#LaRosaetal2020)] | Rome, Italy | WWTP | 250 ml | Centrifugation | Centrifugation | ORF1ab |
|  | [[25](#LaRosaetal2020)] | Italy | WWTP | 250 ml | Centrifugation | PEG and sodium chloride precipitation | ORF1b-nsp14, rdrp, and E gene |
|  | [[26](#Cutrupietal2021)] | Italy | WWTP | 250 ml | Centrifugation | 4 g PEG 8000 and 0.9 g sodium chloride | ORF1ab |
|  | [[27](#Kocamemietal2020)] | Istanbul, Turkey | WWTP | 250 ml | Filtration | Centrifugation | Rdrp gene |
|  | [[28](#Carcerenyetal2021)] | Spain | WWTP | 200 ml | Centrifugation | Aluminium hydroxide adsorption-precipitation | S Gene |
|  | [[29](#Perezetal2021)] | Spain | WWTP | 200 ml | Centrifugation | Aluminium-based adsorption precipitation | N1 |
|  | [[30](#Giraudetal2021)] | Argentina | WWTP | 300 ml | Centrifugation | PEG precipitation | N1 and N2 |
|  | [[31](#Barriosetal2021)] | Argentina | WWTP | 250 ml | Centrifugation | PEG/nacl precipitation | N1 |
|  | [[32](#Medemaetal2020)] | Netherlands | WWTP | 250 ml | Centrifugation | Ultrafiltration | Nucleocapsid (N1, N2, N3) gene and envelope protein (E) |
|  | [[33](#Calderonetal2021)] | Netherlands | WWTP | 50, 100 and 500 ml | Filtration | Filtration on polyethersulfone (PES) membrane and retention on a 1-ml diethylaminoethyl cellulose (DEAE) anion-exchange chromatography (BIA separations, Slovenia) | N gene, S gene and Orf1ab gene |
|  | [[34](#Amerehetal2021)] | Iran | WWTP | 50 ml | Centrifugation | Polyethylene glycol (PEG) precipitation | ORF1ab and N gene |
|  | [[35](#Rafieeetal2021)] | Iran | Sewage manholes | 40 ml | Centrifugation | Polyethylene glycol (PEG) | ORF1ab (FAM channel) and N (ROX channel) gene |
|  | [[36](#Toriietal2020)] | Tokyo metropolis, Japan | WWTP | 50-ml (for UF and EMV) or 40-ml (for PEG) | Filtration | Ultrafiltration after pre-centrifugation (UF), electronegative membrane vortex (EMV), and polyethylene glycol precipitation after pre-centrifugation (PEG). | N1, N2 and N3 |
|  | [[37](#Hataetal2020)] | Japan | WWTP | 100 ml | Centrifugation | Polyethylene glycol precipitation | N2 and N3 |
|  | [[38](#Haramotoetal2020)] | Japan | WWTP | 200 ml | Centrifugation | Electronegative membrane-vortex (EMV) | ORF1a and S gene |
|  | [[39](#Alaminetal2022)] | Japan | WWTP | 40-ml | Centrifugation | Polyethylene glycol precipitation | CDCN1 |
|  | [[40](#Rosilesetal2021)] | Japan | WWTP | 300 ml | Centrifugation | PEG precipitation | CDCN1 and CDC-N2 |
|  | [[41](#Rosilesetal2021)] | Quintana Roo, Mexico | WWTP | 200 ml | Filtration | Polyethylene glycol (PEG) precipitation | N1 and N2 gene |
|  | [[42](#Ahmedetal2021)] | Australia | Suburban pumping station, WWTP | 100–200 ml | Centrifugation | Ultracentrifugation | N_Sarbeco |
|  | [[43](#Yanivetal2021)] | Israel | WWTP | 2 to 5 L | Filtration | Filtration | N1, N2 and N3 |
|  | [[44](#Boogaertsetal2021)] | Belgium | WWTP | - | Ultracentrifugation | PEG 8000 and sodium chloride | N1, N2, N3 and E gene |
|  | [[45](#Mlejnkovaetal2020)] | Czech Republic | WWTP | 500 ml | Centrifugation | Flocculation | - |
|  | [[46](#Sangsanontetal2022)] | Bangkok, Thailand | WWTP | 500–1,000 ml | Centrifugation | Electronegative membrane filtration method with magnesium chloride (mgcl2) | N1 |
|  | [[47](#DAoustetal2021)] | Canada | WRRF | 250 ml | Centrifugation | PEG | CDC N1 and N2 gene |
|  | [[48](#Graberetal2021)] | Canada | WRRF | 40 ml | Centrifugation | Centrifugation | N1 gene |
|  | [[49](#DAoustetal2021)] | Canada | WRRF | 250 ml | Centrifugation | Sodium chloride (nacl)/polyethylene glycol (PEG) | N1 and N2 gene |
|  | [[50](#Corchisetal2021)] | Canada | Municipal sewer system | 250-ml | Centrifugation | Ultrafiltration | N1 |
|  | [[51](#Qiuetal2022)] | Canada | WWTP | 100 ml | Centrifugation | Ultrafiltration | RNA-dependent RNA polymerase (rdrp) gene, E gene, N1 and N2 gene |
|  | [[52](#Ampueroetal2020)] | Chile | WWTP | 42 ml | - | Ultracentrifugation | ORF1ab, S, and N |
|  | [[53](#Gallardoetal2021)] | Southern Chile | Untreated wastewater | 100 ml | Centrifugation | Ultrafiltration | ORF1ab, N gene |
|  | [[54](#Petalaetal2021)] | Greece | WWTP | 200 ml | Centrifugation | Filtration | N and N2 gene |
|  | [[55](#Ahmedetal2021)] | Bangladesh | Sewage waste tank | 50 ml | Centrifugation | Polyethylene glycol (PEG) | ORF1ab, N gene |
|  | [[56](#Rokaetal2021)] | Hungary | WWTP | 250 ml | Centrifugation | Flocculation and ultrafiltration | N1 |

**Table S2.** Primers/Probes used for the amplification of SARS-CoV-2 RNA in wastewater

| Target gene fragment | Primer/ Probe | Sequence | References |
| --- | --- | --- | --- |
| Envelope(E) | E_Sarbeco_F | 5′-ACAGGTACGTTAATAGTTAATAGCGT-3′ | [[57](#Medemaetal2020)][[58](#Wurtzeretal2020)] |
|  | E_Sarbeco_R | 5′-ATATTGCAGCAGTACGCACACA-3′ |  |
|  | E_Sarbeco_P1 | 5′-FAM-ACACTAGCCATCCTTACTGCGCTTCG-ZEN/Iowa Black-3′ |  |
| Nucleocapsid (N) | 2019-nCoV_N1-F | 5′-GACCCCAAAATCAGCGAAAT-3′ | [[59](#Wuetal2020)][[60](#Medemaetal2020)][[61](#Ahmedetal2021)] |
|  | 2019-nCoV_N1-R | 5′-TCTGGTTACTGCCAGTTGAATCTG-3′ |  |
|  | 2019-nCoV_N1-P | 5′-FAM-ACCCCGCATTACGTTTGGTGGACC-ZEN/Iowa Black-3′ |  |
| Nucleocapsid (N) | 2019-nCoV_N2-F | 5′-TTACAAACATTGGCCGCAAA-3′ |  |
|  | 2019-nCoV_N2-R | 5′-GCGCGACATTCCGAAGAA-3′ | [[62](#Wuetal2020)][[63](#Medemaetal2020)] |
|  | 2019-nCoV_N2-P | 5′-FAM-ACAATTTGCCCCCAGCGCTTCAG- ZEN/Iowa Black-3′ |  |
| Nucleocapsid (N) | 2019-nCoV_N3-F | 5′-GGGAGCCTTGAATACACCAAAA-3′ | [[64](#Wuetal2020)][[65](#Medemaetal2020)] |
|  | 2019-nCoV_N3-R | 5′-TGTAGCACGATTGCAGCATTG-3′ |  |
|  | 2019-nCoV_N3-P | 5′-FAM-AYCACATTGGCACCCGCAATCCTG- ZEN/Iowa Black-3′ |  |
| ORF1ab | ORF1ab-F | 5′-CCC TGT GGG TTT TAC ACT TAA-3′ | [[66](#Weietal2020)] |
|  | ORF1ab-R | 5′-ACG ATT GTG CAT CAG CTG A-3′ |  |
|  | ORF1ab-P | 5′-FAM−CCG TCT GCG GTA TGT GGA AAG GTT ATG G−BHQ1-3′ |  |
| S protein | WuhanCoV-spk1-f | 5′-TTGGCAAAATTCAAGA  CTCACTTT-3′ | [[67](#Shiratoetal2020)] |
|  | WuhanCoV-spk2-r | 5′-TGTGGTTCATAAAAAT  TCCTTTGTG-3′ |  |
|  | NIID_WH-1_F24381 | 5′-TCAAGACTCACTTTCT  TCCAC-3′ |  |
|  | NIID_WH-1_R24873 | 5′-ATTTGAAACAAAGACA  CCTTCAC-3′ |  |
| S protein | RBD-qF1 | 5′-CAATGGTTTAACAGGC  ACAGG-3′ | [[68](#Zhaoetal2020)] |
|  | RBD-qR1 | 5′-CTCAAGTGTCTGTGGA  TCACG-3′ |  |
| RdRp | RdRp_SARSr-F | 5′-GTGARATGGTCATGTG  TGGCGG-3′ | [[69](#Cormanetal2020)] |
|  | RdRp_SARSr-R | 5′-CARATGTTAAASACAC  TATTAGCATA-3′ |  |
|  | RdRp_SARSr-P2 | 5′-FAM-CAGGTGGAACC  TCATCAGGAGATG  C-BBQ-3′ |  |
|  | RdRP_SARSr-P1 | 5′-FAM-CCAGGTGG  WACRTCATCMGGTGAT  GC-BBQ-3′ |  |
| RdRp | nCoV_IP2-12669Fw | 5′-ATGAGCTTAGTCCTGT  TG-3′ | [[70](#Kitajimaetal2020)] |
|  | nCoV_IP2-12759Rv | 5′-CTCCCTTTGTTGTGTTGT-3′ |  |
|  | nCoV_IP2-12696bProbe(+) | 5′-HEX-AGATGTCTTGT  GCTGCCGGTA-BHQ1-3′ |  |
| RdRp | nCoV_IP4-14059Fw | 5′-GGTAACTGGTATGATT  TCG-3′ | [[71](#Kitajimaetal2020)] |
|  | nCoV_IP4-14146Rv | 5′-CTGGTCAAGGTTAATA  TAGG-3′ |  |
|  | nCoV_IP4-14084Probe(+) | 5′-FAM-TCATACAAACC  ACGCCAGG-BHQ1-3′ |  |

References

1. Fontenele, Rafaela S., Simona Kraberger, James Hadfield, Erin M. Driver, Devin Bowes, LaRinda A. Holland, Temitope OC Faleye et al. "High-throughput sequencing of SARS-CoV-2 in wastewater provides insights into circulating variants." *Water Research* 205 (2021): 117710.
2. Wu, Fuqing, Jianbo Zhang, Amy Xiao, Xiaoqiong Gu, Wei Lin Lee, Federica Armas, Kathryn Kauffman et al. "SARS-CoV-2 titers in wastewater are higher than expected from clinically confirmed cases." *Msystems* 5, no. 4 (2020): 10-1128.
3. Nemudryi, Artem, Anna Nemudraia, Tanner Wiegand, Kevin Surya, Murat Buyukyoruk, Calvin Cicha, Karl K. Vanderwood, Royce Wilkinson, and Blake Wiedenheft. "Temporal detection and phylogenetic assessment of SARS-CoV-2 in municipal wastewater." *Cell Reports Medicine* 1, no. 6 (2020).
4. LaTurner, Zachary W., David M. Zong, Prashant Kalvapalle, Kiara Reyes Gamas, Austen Terwilliger, Tessa Crosby, Priyanka Ali et al. "Evaluating recovery, cost, and throughput of different concentration methods for SARS-CoV-2 wastewater-based epidemiology." *Water research* 197 (2021): 117043.
5. Ai, Yuehan, Angela Davis, Dan Jones, Stanley Lemeshow, Huolin Tu, Fan He, Peng Ru, Xiaokang Pan, Zuzana Bohrerova, and Jiyoung Lee. "Wastewater SARS-CoV-2 monitoring as a community-level COVID-19 trend tracker and variants in Ohio, United States." *Science of The Total Environment* 801 (2021): 149757.
6. Rainey, Andrew L., Julia C. Loeb, Sarah E. Robinson, John A. Lednicky, John McPherson, Sue Colson, Michael Allen et al. "Wastewater surveillance for SARS-CoV-2 in a small coastal community: Effects of tourism on viral presence and variant identification among low prevalence populations." *Environmental Research* 208 (2022): 112496.
7. Fontenele, Rafaela S., Simona Kraberger, James Hadfield, Erin M. Driver, Devin Bowes, LaRinda A. Holland, Temitope OC Faleye et al. "High-throughput sequencing of SARS-CoV-2 in wastewater provides insights into circulating variants." *Water Research* 205 (2021): 117710.
8. Gregory, Devon A., Chris G. Wieberg, Jeff Wenzel, Chung-Ho Lin, and Marc C. Johnson. "Monitoring SARS-CoV-2 populations in wastewater by amplicon sequencing and using the novel program SAM Refiner." *Viruses* 13, no. 8 (2021): 1647.
9. Kumar, Manish, Shanta Dutta, Siming You, Gang Luo, Shicheng Zhang, Pau Loke Show, Ankush D. Sawarkar, Lal Singh, and Daniel CW Tsang. "A critical review on biochar for enhancing biogas production from anaerobic digestion of food waste and sludge." *Journal of Cleaner Production* 305 (2021): 127143.
10. Kumar, Manish, Arbind Kumar Patel, Anil V. Shah, Janvi Raval, Neha Rajpara, Madhvi Joshi, and Chaitanya G. Joshi. "First proof of the capability of wastewater surveillance for COVID-19 in India through detection of genetic material of SARS-CoV-2." *Science of The Total Environment* 746 (2020): 141326.
11. Dharmadhikari, Tanmay, Rakeshkumar Yadav, Syed Dastager, and Mahesh Dharne. "Translating SARS-CoV-2 wastewater-based epidemiology for prioritizing mass vaccination: a strategic overview." *Environmental Science and Pollution Research* 28 (2021): 42975-42980.
12. Kumar, Manish, Shanta Dutta, Siming You, Gang Luo, Shicheng Zhang, Pau Loke Show, Ankush D. Sawarkar, Lal Singh, and Daniel CW Tsang. "A critical review on biochar for enhancing biogas production from anaerobic digestion of food waste and sludge." *Journal of Cleaner Production* 305 (2021): 127143.
13. Kumar, Manish, Shanta Dutta, Siming You, Gang Luo, Shicheng Zhang, Pau Loke Show, Ankush D. Sawarkar, Lal Singh, and Daniel CW Tsang. "A critical review on biochar for enhancing biogas production from anaerobic digestion of food waste and sludge." *Journal of Cleaner Production* 305 (2021): 127143.
14. Claro, Ieda Carolina Mantovani, Aline Diniz Cabral, Matheus Ribeiro Augusto, Adriana Feliciano Alves Duran, Melissa Cristina Pereira Graciosa, Fernando Luiz Affonso Fonseca, Marcia Aparecida Speranca, and Rodrigo de Freitas Bueno. "Long-term monitoring of SARS-COV-2 RNA in wastewater in Brazil: a more responsive and economical approach." *Water Research* 203 (2021): 117534.
15. Fongaro, Gislaine, Patrícia Hermes Stoco, Doris Sobral Marques Souza, Edmundo Carlos Grisard, Maria Elisa Magri, Paula Rogovski, Marcos André Schörner et al. "The presence of SARS-CoV-2 RNA in human sewage in Santa Catarina, Brazil, November 2019." *Science of The Total Environment* 778 (2021): 146198.
16. Wurtzer, Sébastien, V. Marechal, J. M. Mouchel, Yvon Maday, Remy Teyssou, E. Richard, J. L. Almayrac, and et L. Moulin. "Evaluation of lockdown effect on SARS-CoV-2 dynamics through viral genome quantification in waste water, Greater Paris, France, 5 March to 23 April 2020." *Eurosurveillance* 25, no. 50 (2020): 2000776.
17. Wurtzer, Sebastien, Prunelle Waldman, Audrey Ferrier-Rembert, Gaelle Frenois-Veyrat, Jean-Marie Mouchel, Mickael Boni, Yvon Maday, Vincent Marechal, and Laurent Moulin. "Several forms of SARS-CoV-2 RNA can be detected in wastewaters: implication for wastewater-based epidemiology and risk assessment." *Water Research* 198 (2021): 117183.
18. Agrawal, Shelesh, Laura Orschler, and Susanne Lackner. "Long-term monitoring of SARS-CoV-2 RNA in wastewater of the Frankfurt metropolitan area in Southern Germany." *Scientific reports* 11, no. 1 (2021): 5372.
19. Rubio-Acero, Raquel, Noemi Castelletti, Volker Fingerle, Laura Olbrich, Abhishek Bakuli, Roman Wölfel, Philipp Girl et al. "In search of the SARS-CoV-2 protection correlate: head-to-head comparison of two quantitative S1 assays in pre-characterized oligo-/asymptomatic patients." *Infectious diseases and therapy* 10, no. 3 (2021): 1505-1518.
20. Dumke, Roger, Magali de la Cruz Barron, Reinhard Oertel, Björn Helm, Rene Kallies, Thomas U. Berendonk, and Alexander Dalpke. "Evaluation of two methods to concentrate SARS-CoV-2 from untreated wastewater." *Pathogens* 10, no. 2 (2021): 195.
21. Fitzgerald, Stephen F., Gianluigi Rossi, Alison S. Low, Sean P. McAteer, Brian O’Keefe, David Findlay, Graeme J. Cameron et al. "Site specific relationships between COVID-19 cases and SARS-CoV-2 viral load in wastewater treatment plant influent." *Environmental science & technology* 55, no. 22 (2021): 15276-15286.
22. Hillary, Luke S., Kathryn H. Maher, Anita Lucaci, Jamie Thorpe, Marco A. Distaso, William H. Gaze, Steve Paterson et al. "Monitoring SARS-CoV-2 in municipal wastewater to evaluate the success of lockdown measures for controlling COVID-19 in the UK." *Water Research* 200 (2021): 117214.
23. Martin, Javier, Dimitra Klapsa, Thomas Wilton, Maria Zambon, Emma Bentley, Erika Bujaki, Martin Fritzsche, Ryan Mate, and Manasi Majumdar. "Tracking SARS-CoV-2 in sewage: evidence of changes in virus variant predominance during COVID-19 pandemic." *Viruses* 12, no. 10 (2020): 1144.
24. La Rosa, Giuseppina, Lucia Bonadonna, Luca Lucentini, Sebastien Kenmoe, and Elisabetta Suffredini. "Coronavirus in water environments: Occurrence, persistence and concentration methods-A scoping review." *Water research* 179 (2020): 115899.
25. La Rosa, Giuseppina, Lucia Bonadonna, Luca Lucentini, Sebastien Kenmoe, and Elisabetta Suffredini. "Coronavirus in water environments: Occurrence, persistence and concentration methods-A scoping review." *Water research* 179 (2020): 115899.
26. Cutrupi, F., M. Cadonna, S. Manara, and P. Foladori. "Surveillance of SARS-CoV-2 in extensive monitoring of municipal wastewater: key issues to yield reliable results." *Water Science and Technology* 84, no. 12 (2021): 3508-3514.
27. Kocamemi, Bilge Alpaslan, Halil Kurt, Ahmet Sait, Fahriye Sarac, Ahmet Mete Saatci, and Bekir Pakdemirli. "SARS-CoV-2 detection in Istanbul wastewater treatment plant sludges." *MedRxiv* (2020): 2020-05.
28. Carcereny, Albert, Adán Martínez-Velázquez, Albert Bosch, Ana Allende, Pilar Truchado, Jenifer Cascales, Jesús L. Romalde et al. "Monitoring emergence of the SARS-CoV-2 B. 1.1. 7 variant through the Spanish national SARS-CoV-2 wastewater surveillance system (VATar COVID-19)." *Environmental science & technology* 55, no. 17 (2021): 11756-11766.
29. Pérez-Cataluña, Alba, Enric Cuevas-Ferrando, Walter Randazzo, Irene Falco, Ana Allende, and Gloria Sanchez. "Comparing analytical methods to detect SARS-CoV-2 in wastewater." *Science of the Total Environment* 758 (2021): 143870.
30. Giraud-Billoud, Maximiliano, Paula Cuervo, Jorgelina C. Altamirano, Marcela Pizarro, Julieta N. Aranibar, Adolfo Catapano, Héctor Cuello, Gisela Masachessi, and Israel A. Vega. "Monitoring of SARS-CoV-2 RNA in wastewater as an epidemiological surveillance tool in Mendoza, Argentina." *Science of the Total Environment* 796 (2021): 148887.
31. Barrios, Melina Elizabeth, Sofía Micaela Díaz, Carolina Torres, Damián Matías Costamagna, María Dolores Blanco Fernández, and Viviana Andrea Mbayed. "Dynamics of SARS-CoV-2 in wastewater in three districts of the Buenos Aires metropolitan region, Argentina, throughout nine months of surveillance: A pilot study." *Science of the Total Environment* 800 (2021): 149578.
32. Medema, Gertjan, Leo Heijnen, Goffe Elsinga, Ronald Italiaander, and Anke Brouwer. "Presence of SARS-Coronavirus-2 RNA in sewage and correlation with reported COVID-19 prevalence in the early stage of the epidemic in the Netherlands." *Environmental Science & Technology Letters* 7, no. 7 (2020): 511-516.
33. Calderon-Franco, David, Seeram Apoorva, Gertjan Medema, Mark CM van Loosdrecht, and David G. Weissbrodt. "Upgrading residues from wastewater and drinking water treatment plants as low-cost adsorbents to remove extracellular DNA and microorganisms carrying antibiotic resistance genes from treated effluents." *Science of the Total Environment* 778 (2021): 146364.
34. Amereh, Fatemeh, Masoud Negahban-Azar, Siavash Isazadeh, Hossein Dabiri, Najmeh Masihi, Mahsa Jahangiri-Rad, and Mohammad Rafiee. "Sewage systems surveillance for SARS-CoV-2: identification of knowledge gaps, emerging threats, and future research needs." *Pathogens* 10, no. 8 (2021): 946.
35. Rafiee, Mohammad, Siavash Isazadeh, Anoushiravan Mohseni-Bandpei, Seyed Reza Mohebbi, Mahsa Jahangiri-Rad, Akbar Eslami, Hossein Dabiri, Kasra Roostaei, Mohammad Tanhaei, and Fatemeh Amereh. "Moore swab performs equal to composite and outperforms grab sampling for SARS-CoV-2 monitoring in wastewater." *Science of The Total Environment* 790 (2021): 148205.
36. Torii, Shotaro, Masae Itamochi, and Hiroyuki Katayama. "Inactivation kinetics of waterborne virus by ozone determined by a continuous quench flow system." *Water Research* 186 (2020): 116291.
37. Hata, Akihiko, and Ryo Honda. "Potential sensitivity of wastewater monitoring for SARS-CoV-2: comparison with norovirus cases." (2020): 6451-6452.
38. Haramoto, Eiji, Bikash Malla, Ocean Thakali, and Masaaki Kitajima. "First environmental surveillance for the presence of SARS-CoV-2 RNA in wastewater and river water in Japan." *Science of the Total Environment* 737 (2020): 140405.
39. Alamin, Md, Shohei Tsuji, Akihiko Hata, Hiroe Hara-Yamamura, and Ryo Honda. "Selection of surrogate viruses for process control in detection of SARS-CoV-2 in wastewater." *Science of The Total Environment* 823 (2022): 153737.
40. Rosiles-González, Gabriela, Victor Hugo Carrillo-Jovel, Liliana Alzate-Gaviria, Walter Q. Betancourt, Charles P. Gerba, Oscar A. Moreno-Valenzuela, Raúl Tapia-Tussell, and Cecilia Hernández-Zepeda. "Environmental surveillance of SARS-CoV-2 RNA in wastewater and groundwater in Quintana Roo, Mexico." *Food and Environmental Virology* 13, no. 4 (2021): 457-469.
41. Rosiles-González, Gabriela, Victor Hugo Carrillo-Jovel, Liliana Alzate-Gaviria, Walter Q. Betancourt, Charles P. Gerba, Oscar A. Moreno-Valenzuela, Raúl Tapia-Tussell, and Cecilia Hernández-Zepeda. "Environmental surveillance of SARS-CoV-2 RNA in wastewater and groundwater in Quintana Roo, Mexico." *Food and Environmental Virology* 13, no. 4 (2021): 457-469.
42. Ahmed, Firoz, Md Aminul Islam, Manish Kumar, Maqsud Hossain, Prosun Bhattacharya, Md Tahmidul Islam, Foysal Hossen et al. "First detection of SARS-CoV-2 genetic material in the vicinity of COVID-19 isolation Centre in Bangladesh: Variation along the sewer network." *Science of the total environment* 776 (2021): 145724.
43. Yaniv, Karin, Eden Ozer, Marilou Shagan, Satish Lakkakula, Noam Plotkin, Nikhil Suresh Bhandarkar, and Ariel Kushmaro. "Direct RT-qPCR assay for SARS-CoV-2 variants of concern (Alpha, B. 1.1. 7 and Beta, B. 1.351) detection and quantification in wastewater." *Environmental Research* 201 (2021): 111653.
44. Boogaerts, Tim, Fahad Ahmed, Phil M. Choi, Benjamin Tscharke, Jake O'Brien, Hans De Loof, Jianfa Gao et al. "Current and future perspectives for wastewater-based epidemiology as a monitoring tool for pharmaceutical use." *Science of the Total Environment* 789 (2021): 148047.
45. Mlejnkova, Hana, Katerina Sovova, Petra Vasickova, Vera Ocenaskova, Lucie Jasikova, and Eva Juranova. "Preliminary study of Sars-Cov-2 occurrence in wastewater in the Czech Republic." *International journal of environmental research and public health* 17, no. 15 (2020): 5508.
46. Sangsanont, Jatuwat, Surapong Rattanakul, Akechai Kongprajug, Natcha Chyerochana, Montakarn Sresung, Nonnarit Sriporatana, Nasamon Wanlapakorn, Yong Poovorawan, Skorn Mongkolsuk, and Kwanrawee Sirikanchana. "SARS-CoV-2 RNA surveillance in large to small centralized wastewater treatment plants preceding the third COVID-19 resurgence in Bangkok, Thailand." *Science of The Total Environment* 809 (2022): 151169.
47. D'Aoust, Patrick M., Syeda Tasneem Towhid, Élisabeth Mercier, Nada Hegazy, Xin Tian, Kamya Bhatnagar, Zhihao Zhang et al. "COVID-19 wastewater surveillance in rural communities: Comparison of lagoon and pumping station samples." *Science of the Total Environment* 801 (2021): 149618.
48. Graber, Tyson E., Élisabeth Mercier, Kamya Bhatnagar, Meghan Fuzzen, Patrick M. D'Aoust, Huy-Dung Hoang, Xin Tian et al. "Near real-time determination of B. 1.1. 7 in proportion to total SARS-CoV-2 viral load in wastewater using an allele-specific primer extension PCR strategy." *Water research* 205 (2021): 117681.
49. D'Aoust, Patrick M., Syeda Tasneem Towhid, Élisabeth Mercier, Nada Hegazy, Xin Tian, Kamya Bhatnagar, Zhihao Zhang et al. "COVID-19 wastewater surveillance in rural communities: Comparison of lagoon and pumping station samples." *Science of the Total Environment* 801 (2021): 149618.
50. Corchis-Scott, Ryland, Qiudi Geng, Rajesh Seth, Rajan Ray, Mohsan Beg, Nihar Biswas, Lynn Charron et al. "Averting an outbreak of SARS-CoV-2 in a university residence hall through wastewater surveillance." *Microbiology spectrum* 9, no. 2 (2021): e00792-21.
51. Qiu, Yuanyuan, Jiaao Yu, Kanti Pabbaraju, Bonita E. Lee, Tiejun Gao, Nicholas J. Ashbolt, Steve E. Hrudey et al. "Validating and optimizing the method for molecular detection and quantification of SARS-CoV-2 in wastewater." *Science of the Total Environment* 812 (2022): 151434.
52. Ampuero, Manuel, Santiago Valenzuela, Fernando Valiente-Echeverría, Ricardo Soto-Rifo, Gonzalo P. Barriga, Jonás Chnaiderman, Cecilia Rojas, Sergio Guajardo-Leiva, Beatriz Díez, and Aldo Gaggero. "SARS-CoV-2 detection in sewage in Santiago, Chile-preliminary results." *MedRxiv* (2020): 2020-07.
53. Gallardo-Escárate, Cristian, Valentina Valenzuela-Muñoz, Gustavo Núñez-Acuña, Diego Valenzuela-Miranda, Bárbara P. Benaventel, Constanza Sáez-Vera, Homero Urrutia et al. "The wastewater microbiome: a novel insight for COVID-19 surveillance." *Science of The Total Environment* 764 (2021): 142867.
54. Petala, Maria, D. Dafou, M. Kostoglou, Th Karapantsios, E. Kanata, A. Chatziefstathiou, F. Sakaveli et al. "A physicochemical model for rationalizing SARS-CoV-2 concentration in sewage. Case study: The city of Thessaloniki in Greece." *Science of The Total Environment* 755 (2021): 142855.
55. Ahmed, Firoz, Md Aminul Islam, Manish Kumar, Maqsud Hossain, Prosun Bhattacharya, Md Tahmidul Islam, Foysal Hossen et al. "First detection of SARS-CoV-2 genetic material in the vicinity of COVID-19 isolation Centre in Bangladesh: Variation along the sewer network." *Science of the total environment* 776 (2021): 145724.
56. Róka, Eszter, Bernadett Khayer, Zoltán Kis, Luca Bella Kovács, Eszter Schuler, Nóra Magyar, Tibor Málnási et al. "Ahead of the second wave: Early warning for COVID-19 by wastewater surveillance in Hungary." *Science of the Total Environment* 786 (2021): 147398.
57. Medema, Gertjan, Leo Heijnen, Goffe Elsinga, Ronald Italiaander, and Anke Brouwer. "Presence of SARS-Coronavirus-2 RNA in sewage and correlation with reported COVID-19 prevalence in the early stage of the epidemic in the Netherlands." *Environmental Science & Technology Letters* 7, no. 7 (2020): 511-516.
58. Wurtzer, Sébastien, V. Marechal, J. M. Mouchel, Yvon Maday, Remy Teyssou, E. Richard, J. L. Almayrac, and et L. Moulin. "Evaluation of lockdown effect on SARS-CoV-2 dynamics through viral genome quantification in waste water, Greater Paris, France, 5 March to 23 April 2020." *Eurosurveillance* 25, no. 50 (2020): 2000776.
59. Wu, Fuqing, Jianbo Zhang, Amy Xiao, Xiaoqiong Gu, Wei Lin Lee, Federica Armas, Kathryn Kauffman et al. "SARS-CoV-2 titers in wastewater are higher than expected from clinically confirmed cases." *Msystems* 5, no. 4 (2020): 10-1128.
60. Medema, Gertjan, Leo Heijnen, Goffe Elsinga, Ronald Italiaander, and Anke Brouwer. "Presence of SARS-Coronavirus-2 RNA in sewage and correlation with reported COVID-19 prevalence in the early stage of the epidemic in the Netherlands." *Environmental Science & Technology Letters* 7, no. 7 (2020): 511-516.
61. Ahmed, Firoz, Md Aminul Islam, Manish Kumar, Maqsud Hossain, Prosun Bhattacharya, Md Tahmidul Islam, Foysal Hossen et al. "First detection of SARS-CoV-2 genetic material in the vicinity of COVID-19 isolation Centre in Bangladesh: Variation along the sewer network." *Science of the total environment* 776 (2021): 145724.
62. Wu, Fuqing, Jianbo Zhang, Amy Xiao, Xiaoqiong Gu, Wei Lin Lee, Federica Armas, Kathryn Kauffman et al. "SARS-CoV-2 titers in wastewater are higher than expected from clinically confirmed cases." *Msystems* 5, no. 4 (2020): 10-1128.
63. Medema, Gertjan, Leo Heijnen, Goffe Elsinga, Ronald Italiaander, and Anke Brouwer. "Presence of SARS-Coronavirus-2 RNA in sewage and correlation with reported COVID-19 prevalence in the early stage of the epidemic in the Netherlands." *Environmental Science & Technology Letters* 7, no. 7 (2020): 511-516.
64. Wu, Fuqing, Jianbo Zhang, Amy Xiao, Xiaoqiong Gu, Wei Lin Lee, Federica Armas, Kathryn Kauffman et al. "SARS-CoV-2 titers in wastewater are higher than expected from clinically confirmed cases." *Msystems* 5, no. 4 (2020): 10-1128.
65. Medema, Gertjan, Leo Heijnen, Goffe Elsinga, Ronald Italiaander, and Anke Brouwer. "Presence of SARS-Coronavirus-2 RNA in sewage and correlation with reported COVID-19 prevalence in the early stage of the epidemic in the Netherlands." *Environmental Science & Technology Letters* 7, no. 7 (2020): 511-516.
66. Wei, Wycliffe E., Zongbin Li, Calvin J. Chiew, Sarah E. Yong, Matthias P. Toh, and Vernon J. Lee. "Presymptomatic transmission of SARS-CoV-2—Singapore, january 23–march 16, 2020." *Morbidity and Mortality Weekly Report* 69, no. 14 (2020): 411.
67. Shirato, Kazuya, Naganori Nao, Harutaka Katano, Ikuyo Takayama, Shinji Saito, Fumihiro Kato, Hiroshi Katoh et al. "Development of genetic diagnostic methods for detection for novel coronavirus 2019 (nCoV-2019) in Japan." *Japanese journal of infectious diseases* 73, no. 4 (2020): 304-307.
68. Zhao, Bing, Chao Ni, Ran Gao, Yuyan Wang, Li Yang, Jinsong Wei, Ting Lv et al. "Recapitulation of SARS-CoV-2 infection and cholangiocyte damage with human liver ductal organoids." *Protein & cell* 11, no. 10 (2020): 771-775.
69. Corman, Victor M., Holger F. Rabenau, Ortwin Adams, Doris Oberle, Markus B. Funk, Brigitte Keller‐Stanislawski, Jörg Timm, Christian Drosten, and Sandra Ciesek. "SARS‐CoV‐2 asymptomatic and symptomatic patients and risk for transfusion transmission." *Transfusion* 60, no. 6 (2020): 1119.
70. Kitajima, Masaaki, Warish Ahmed, Kyle Bibby, Annalaura Carducci, Charles P. Gerba, Kerry A. Hamilton, Eiji Haramoto, and Joan B. Rose. "SARS-CoV-2 in wastewater: State of the knowledge and research needs." *Science of The Total Environment* 739 (2020): 139076.
71. Kitajima, Masaaki, Warish Ahmed, Kyle Bibby, Annalaura Carducci, Charles P. Gerba, Kerry A. Hamilton, Eiji Haramoto, and Joan B. Rose. "SARS-CoV-2 in wastewater: State of the knowledge and research needs." *Science of The Total Environment* 739 (2020): 139076.
